# Serum Uric Acid as a Biomarker for Incident Type 2 Diabetes Mellitus: A 6-Year Cohort Study in Qatar

**DOI:** 10.3390/metabo16040251

**Published:** 2026-04-08

**Authors:** Alan Saeed, Yamane Chawa, Samer Kaspo, Hassan Ibrahim, Aisha Al Adab, Anas Kalfah

**Affiliations:** 1 Primary Health Care Corporation, Doha P.O. Box 26555, Qatar; ankalfah@phcc.gov.qa; 2 Hamad Medical Corporation, Doha P.O. Box 3050, Qatar; ychawa@hamad.qa (Y.C.); hibrahim16@hamad.qa (H.I.); aaladab@hamad.qa (A.A.A.); 3 Saint Francis Hospital, Evanston, IL 60202, USA; skaspo@primehealthcare.com

**Keywords:** serum uric acid, hyperuricemia, type 2 diabetes, retrospective cohort, Cox regression, restricted cubic splines, explainable boosting machine, Qatar, sex differences

## Abstract

Background: Serum uric acid (SUA) may predict incident type 2 diabetes mellitus (T2DM), but longitudinal evidence from Middle Eastern populations remains limited. Methods: We conducted a retrospective cohort study using electronic health records from Qatar’s Primary Health Care Corporation over a six-year period (2018–2023). Adults aged ≥18 years with at least one valid serum uric acid (SUA) measurement and no prior diabetes at baseline were eligible. All eligible participants were retained; no propensity score matching was performed. Baseline SUA was defined at the first valid measurement, and repeated-measure exposures included current SUA, cumulative-average SUA, and landmark time-weighted average (TWA) SUA. Sex-specific SUA categories were low <208, normal 208–428, and high >428 µmol/L in males and low <149, normal 149–357, and high >357 µmol/L in females. Sex-stratified Cox models, restricted cubic spline analyses, prespecified sensitivity analyses, and complementary explainable boosting machine (EBM) models were used to evaluate associations with incident type 2 diabetes mellitus (T2DM). Results: The cohort included 169,876 adults (85,361 males and 84,515 females) and 18,714 incident T2DM events. In fully adjusted baseline Cox models, high baseline SUA was associated with higher T2DM hazard in females (hazard ratio [HR]: 1.44; 95% CI: 1.36–1.53), whereas low baseline SUA was associated with higher hazard in males (HR: 1.60; 95% CI: 1.44–1.78), and high SUA was not. In women, positive SUA–T2DM associations persisted in time-varying and landmark analyses, including current high- versus- normal SUA (HR: 1.50; 95% CI: 1.41–1.58) and 2-measurement landmark TWA SUA per 1 mg/dL (HR: 1.17; 95% CI: 1.13–1.20). In men, unlagged whole-cohort analyses showed inverse continuous associations, but lagged and repeated-measure analyses shifted toward positive associations, including 365-day lagged high- versus- normal baseline SUA (HR: 1.19; 95% CI: 1.11–1.28) and 2-measurement landmark TWA SUA per 1 mg/dL (HR: 1.06; 95% CI: 1.03–1.09). Restricted cubic splines showed a steadily rising risk gradient in females above approximately 262 µmol/L and a J-shaped pattern in males, with the lowest risk near 374 µmol/L. In EBM models, TWA SUA ranked third in women and fifth in men in the 2-measurement landmark cohorts. Conclusions: In this large Qatar cohort, longitudinal SUA was associated with incident T2DM in a sex-specific manner, with consistent positive associations in females and exposure-definition-dependent patterns in males. Repeated SUA measurements may improve diabetes risk stratification, but causal and therapeutic implications require further study.

## 1. Introduction

Type 2 diabetes mellitus (T2DM) is a major global health challenge associated with substantial morbidity, premature mortality, and health-system costs. The International Diabetes Federation (IDF) estimated that 537 million adults were living with diabetes in 2021 (approximately 1 in 10 adults), with >90% representing T2DM; projections indicate continued growth to 643 million by 2030 and 783 million by 2045 [1,2].

In Qatar, the diabetes burden is particularly high. IDF estimates indicate that approximately 16% of adults were living with diabetes in 2021, and local modeling studies suggest that the prevalence could increase substantially over the coming decades if current risk-factor patterns persist [3,4]. These trends underscore the need for earlier identification of high-risk individuals and scalable prevention strategies in routine care.

Hyperuricemia—typically defined as elevated serum uric acid (SUA)—has emerged as a potentially important and modifiable cardiometabolic risk marker. SUA is the final product of purine metabolism and may exert antioxidant effects at physiologic concentrations; however, at higher levels, uric acid may shift toward pro-oxidant behavior, promoting oxidative stress, endothelial dysfunction, and inflammatory signaling, pathways closely linked to insulin resistance and β-cell dysfunction [5,6,7]. Elevated SUA also clusters with cardiometabolic conditions such as hypertension and metabolic syndrome, further supporting its relevance to diabetes risk biology [8,9].

Epidemiologic evidence supports SUA as an early predictor of dysglycemia and incident T2DM. Prospective cohort studies demonstrate that higher baseline SUA precedes and predicts future T2DM, and meta-analyses consistently show increased diabetes risk with higher SUA after adjustment for conventional risk factors [10,11,12]. Longitudinal analyses further suggest that cumulative or persistent SUA exposure may be particularly relevant to diabetes development [13]. In addition, sex-specific analyses indicate that women may experience increasing dysglycemic risk at lower SUA levels than men, which is consistent with differences in urate handling and hormonal milieu [14].

Despite growing international evidence, data from Middle Eastern populations remain limited, and few studies have leveraged repeated SUA measurements within a true longitudinal cohort framework. Using electronic health records from Qatar’s Primary Health Care Corporation, we conducted a retrospective cohort study to: (i) quantify sex-specific associations between SUA and incident T2DM using baseline, time-varying, lagged, and landmark Cox models; (ii) characterize nonlinear dose–response patterns using restricted cubic splines; and (iii) evaluate the relative predictive contribution of longitudinal SUA using interpretable machine learning (explainable boosting machines [EBM]).

## 2. Materials and Methods

### 2.1. Study Design and Data Source

This was a retrospective cohort study assessing the association between serum uric acid (SUA) and incident type 2 diabetes mellitus (T2DM). We used routinely collected electronic health records (EHR) from Qatar’s Primary Health Care Corporation (PHCC) over a fixed six-year study window from 1 January 2018 through to 31 December 2023.

### 2.2. Study Population and Follow-Up

Eligible participants were adults (≥18 years) with at least one valid SUA measurement recorded in PHCC’s EHR system and no evidence of diabetes before baseline. Baseline was defined as the first valid SUA measurement during the study window. Incident T2DM was defined using the diabetes indicator together with the first recorded diabetes diagnosis date after baseline and no later than study end. Follow-up began at baseline and ended at the first T2DM diagnosis, the end of available observation, or 31 December 2023, whichever came first. All eligible participants were retained; no case–control sampling or propensity score matching was performed.

### 2.3. Serum Uric Acid Processing and Exposure Definitions

The source files contained up to 42 SUA measurements per participant, each paired with a laboratory date. Valid SUA measurements were required to be numeric values > 0 with non-missing dates within the study window. When more than one SUA value occurred for the same participant on the same date–time stamp, values were averaged to create a single record for that participant–date. Baseline SUA was analyzed both categorically and continuously, and additional longitudinal exposure definitions included time-varying current SUA, time-varying cumulative-average SUA, and landmark time-weighted average (TWA) SUA calculated using the trapezoidal rule from measurements observed up to the landmark date.

### 2.4. Sex-Specific SUA Categories

The primary categorical exposure used sex-specific clinical thresholds, with the normal category serving as the reference group in all categorical models:

- Males: low (<208 µmol/L), normal (208–428 µmol/L), and high (>428 µmol/L).
- Females: low (<149 µmol/L), normal (149–357 µmol/L), and high (>357 µmol/L).

Continuous analyses expressed SUA per 1 mg/dL increase. For landmark analyses, participants were required to have at least 2 or at least 3 follow-up SUA measurements before the landmark, and post-landmark events were counted only after delayed entry at that landmark date.

### 2.5. Covariates and Statistical Analyses

All multivariable models adjusted for age, baseline calendar year, liver disorder, dyslipidemia, obesity, chronic kidney disease, prediabetes, beta-blocker use, statin use, and allopurinol use. Because the observed associations were sex-specific, all inferential models were fit separately for females and males. Descriptive statistics were summarized by sex and by baseline SUA category using means with standard deviations for continuous variables, medians with interquartile ranges for skewed follow-up variables, and counts with percentages for categorical variables.

The main baseline analyses used Cox proportional hazards regression with Efron handling of tied event times. Fully adjusted models were fit for baseline categorical SUA and for baseline SUA as a continuous exposure per 1 mg/dL. Nonlinear baseline associations were evaluated using sex-stratified restricted cubic spline Cox models plotted over the first-to-ninety-ninth percentile range and referenced to the median SUA within the sex-specific normal category. For descriptive visualization of unadjusted event-free survival, Kaplan–Meier curves were also plotted by sex-specific baseline SUA categories.

Longitudinal analyses used counting-process start–stop Cox models with participant-clustered robust standard errors for time-varying current SUA and cumulative-average SUA. Prespecified sensitivity analyses repeated the baseline Cox models after excluding allopurinol users, after excluding participants with chronic kidney disease, without adjustment for prediabetes, after applying 90-day and 365-day exposure lags, and after restricting to participants with repeated follow-up measurements. Landmark analyses with delayed entry evaluated TWA SUA in cohorts requiring at least 2 or 3 follow-up SUA measurements. Exploratory joint longitudinal–survival models were also attempted among participants with at least 4 follow-up SUA measurements.

### 2.6. Explainable Boosting Machine Analysis

To complement the survival models, we fitted explainable boosting machine (EBM) models in Python (version 3.11) to predict incident T2DM within a fixed 2-year post-landmark window. Separate models were developed for females and males with ≥2 or ≥3 measurements. The landmark was the second or third SUA measurement, respectively. TWA SUA up to the landmark was the primary feature and was winsorized within each sex–scenario combination at the first and ninety-ninth percentiles; only landmarks with a full 2-year follow-up opportunity were eligible.

Model features were TWA SUA, age, liver disorder, dyslipidemia, obesity, chronic kidney disease, prediabetes, beta-blocker use, statin use, and allopurinol use. The primary EBM deliberately excluded the raw calendar year to avoid calendar-time artifacts. Main-effect-only EBMs were trained using an 80/20 stratified train–test split with random state 42, and model performance was summarized using ROC AUC, average precision, Brier score, and log loss. Relative term importance and partial-dependence plots were used to assess the predictive contribution and shape of the TWA SUA term.

### 2.7. Statistical Software and Ethical Approval

All survival analyses were performed in R (version 4.4.0), and the EBM analyses were performed in Python (version 3.11). Proportional hazards were assessed using Schoenfeld residual tests. Ethical approval for this study was obtained from PHCC’s Institutional Review Board, and the study was registered according to local regulations. Patient confidentiality and data privacy were maintained throughout the study.

## 3. Results

The analytic population comprised 169,876 participants: 84,515 females and 85,361 males. During follow-up, 18,714 participants developed incident T2DM. Median follow-up was 3.00 years in females and 3.13 years in males, with maximum follow-up approaching 6 years in both sexes. Repeated SUA measurement density remained substantial: 34,701 females and 38,277 males had at least two follow-up SUA measurements, while 15,374 females and 19,468 males had at least three. Baseline mean SUA was 279.39 µmol/L in females and 367.07 µmol/L in males. In females, crude incident T2DM proportions increased from 5.75% in the low-SUA category to 8.60% in the normal category and 16.95% in the high category. In males, crude incidence was highest in the low-SUA category (22.21%) and similar in the normal (12.11%) and high (12.39%) categories (Table 1).

In fully adjusted baseline Cox models, the association between SUA and incident T2DM differed markedly by sex. In females, high baseline SUA was associated with a higher hazard of incident T2DM compared with normal SUA (HR: 1.44; 95% CI: 1.36–1.53; *p* < 0.001), whereas low SUA was not significantly associated with risk (HR: 0.84; 95% CI: 0.67–1.06; *p* = 0.137). On the continuous scale, each 1 mg/dL increase in baseline SUA was associated with a 16% higher T2DM hazard (HR: 1.16; 95% CI 1.14–1.18; *p* < 0.001). In contrast, male baseline models showed no association for high-versus-normal SUA (HR: 1.00; 95% CI: 0.95–1.06; *p* = 0.887), but low baseline SUA was associated with higher hazard (HR: 1.60; 95% CI: 1.44–1.78; *p* < 0.001). The male continuous baseline model was inversely associated (HR: 0.96 per 1 mg/dL; 95% CI: 0.94–0.97; *p* < 0.001) (Table 2). Descriptive Kaplan–Meier curves stratified by sex-specific baseline SUA categories are shown in Supplementary Figure S1 and were directionally consistent with the adjusted baseline findings: in females, diabetes-free survival was lowest in the high-SUA category, whereas in males, the low-SUA category showed the poorest unadjusted survival.

Time-varying analyses reproduced a consistent positive female pattern. In females, high current SUA versus normal was associated with higher T2DM hazard (HR: 1.50; 95% CI: 1.41–1.58; *p* < 0.001), and the continuous current-SUA model showed HR 1.17 per 1 mg/dL (95% CI: 1.15–1.19; *p* < 0.001); cumulative-average SUA produced nearly identical estimates. In males, the unlagged whole-cohort time-varying models again showed no excess hazard for high SUA and a higher hazard for low SUA, with inverse continuous associations. However, prespecified sensitivity analyses clarified that this male low-SUA pattern was not stable under more conservative exposure definitions. After 90-day and 365-day lagging, high baseline SUA became positively associated with incident T2DM in males (365-day lag HR: 1.19; 95% CI: 1.11–1.28; *p* < 0.001), whereas the low-SUA association attenuated. Similarly, when analyses were restricted to males with repeated follow-up measurements, high SUA became positively associated in the ≥2-measurement cohort (HR: 1.15; 95% CI: 1.06–1.26; *p* = 0.001). By contrast, the female high-SUA association remained positive across all sensitivity analyses.

**Figure 1:**
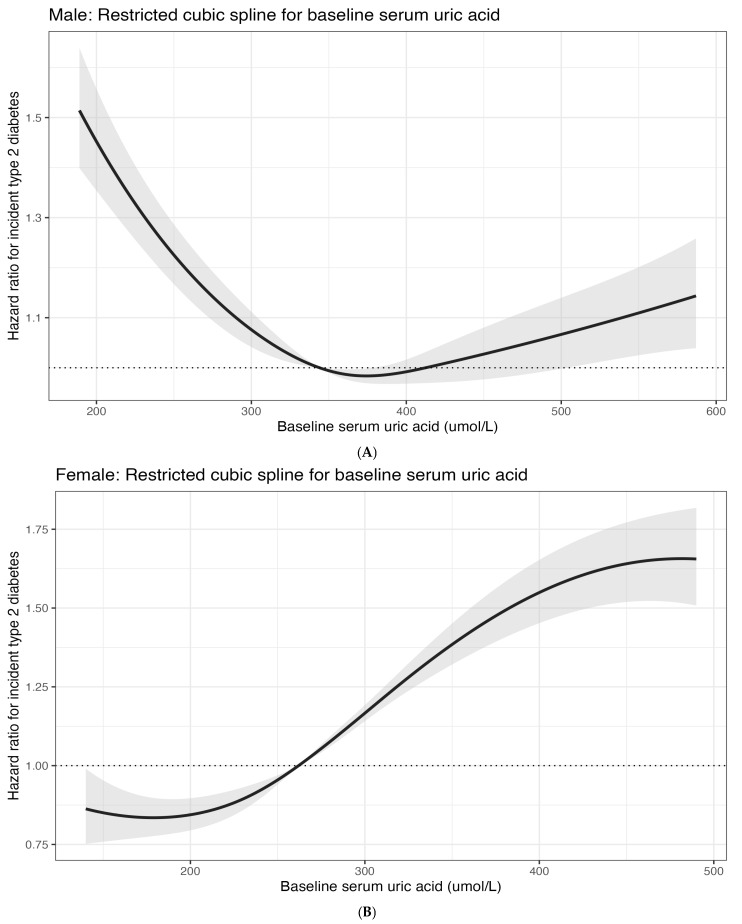
(**A**) Male. Restricted cubic spline Cox curve for baseline serum uric acid and incident type 2 diabetes. (**B**) Female. Restricted cubic spline Cox curve for baseline serum uric acid and incident type 2 diabetes. In both panels, the solid black line shows the adjusted hazard ratio, the gray shaded band shows the 95% confidence interval, and the horizontal dotted line marks HR = 1.0.

Flexible spline analyses reinforced the sex-specific difference in shape (Figure 1). In females, the adjusted spline curve had a nadir near 179 µmol/L, crossed the null at approximately 262 µmol/L, and then rose progressively across higher SUA levels. In males, the baseline spline was J-shaped, with the lowest estimated hazard near 374 µmol/L and approximate null crossings near 344 and 412 µmol/L, indicating higher risk at both low and high extremes relative to the mid-range. Landmark TWA analyses were directionally concordant with these patterns. In the 2-measurement landmark cohort, high-versus-normal TWA SUA was associated with higher post-landmark T2DM hazard in females (HR: 1.41; 95% CI: 1.28–1.55; *p* < 0.001), and the continuous female TWA model showed an HR of 1.17 per 1 mg/dL (95% CI: 1.13–1.20; *p* < 0.001). In males, categorical high-versus-normal TWA estimates were not significant, but the continuous TWA association was positive in the 2-measurement cohort (HR: 1.06 per 1 mg/dL; 95% CI: 1.03–1.09; *p* < 0.001) and borderline positive in the 3-measurement cohort (HR: 1.05; 95% CI: 1.00–1.10; *p* = 0.054).

**Figure 2:**
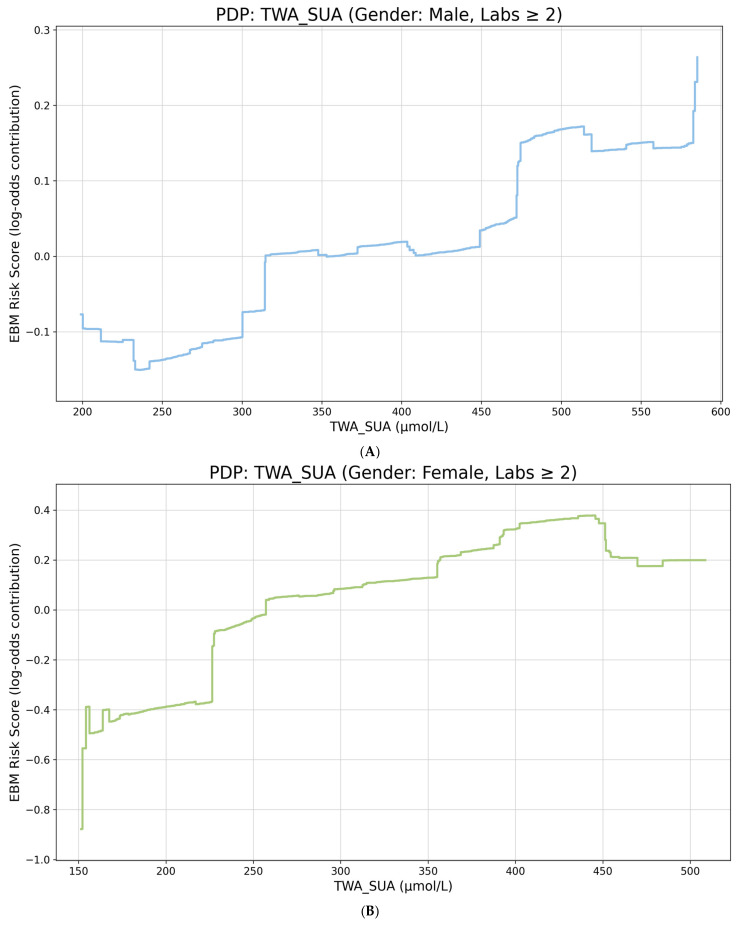
(**A**) Male. EBM partial-dependence plot for landmark TWA SUA and 2-year post-landmark T2DM risk. (**B**) Female. EBM partial-dependence plot for landmark TWA SUA and 2-year post-landmark T2DM risk. In both panels, the line shows the model-based contribution of TWA SUA to the predicted log-odds of post-landmark T2DM after averaging over the other model features; higher values indicate a greater positive contribution to predicted risk.

The EBM analysis evaluated fixed 2-year post-landmark risk and therefore analyzed smaller cohorts than the survival models because participants with landmarks occurring too late to allow a complete prediction horizon were excluded by design. In the 2-measurement EBM scenario, 17,456 females and 21,757 males remained, with hold-out ROC AUC values of 0.739 and 0.651, respectively. In the 3-measurement scenario, the corresponding analytic cohorts were 6368 females and 9261 males, with ROC AUC values of 0.661 and 0.662. Across the four EBM scenarios, TWA SUA ranked third and fourth in females and fifth and eighth in males, indicating a stronger predictive contribution in women. The 2-measurement partial-dependence curves showed that the contribution of TWA SUA crossed from negative to positive around 257 µmol/L in females and began shifting upward around 315–350 µmol/L in males (Figure 2), Key sex-specific effect estimates across the baseline, time-varying, lagged, and landmark analyses are summarized in Table 3.

In male participants, the spline curve showed a J-shaped association between baseline SUA and incident T2DM after adjustment for age, baseline calendar year, liver disorder, dyslipidemia, obesity, chronic kidney disease, prediabetes, beta-blocker use, statin use, and allopurinol use. The curve was referenced to the median baseline SUA within the male normal category and is displayed over the first-to-ninety-ninth percentile range. The lowest estimated hazard occurred near 374 µmol/L, with approximate null crossings at 344 and 412 µmol/L.

In female participants, the adjusted spline curve showed a steadily rising hazard gradient at higher baseline SUA levels. The curve was referenced to the median baseline SUA within the female normal category and is displayed over the first-to-ninety-ninth percentile range. The nadir occurred near 179 µmol/L, and the curve crossed the null at approximately 262 µmol/L before increasing progressively across the upper tail.

In males with at least two qualifying measurements, the EBM partial-dependence plot showed the model-based log-odds contribution of TWA SUA to 2-year post-landmark T2DM risk after averaging over the joint distribution of the other features. The male curve was relatively flat through much of the mid-range and increased more clearly at higher TWA SUA levels, with the strongest positive contribution in the upper tail.

In females, the 2-measurement EBM curve showed its lowest contribution at approximately 152 µmol/L and then rose progressively, with a clear positive contribution above the mid-200 µmol/L range. This pattern was directionally consistent with the female landmark Cox models and supported a relatively low threshold at which higher longitudinal SUA became informative for future diabetes risk.

In males, the 2-measurement EBM curve reached its lowest contribution near 236 µmol/L and then increased more gradually than in females, with a later upward shift in the low-to-mid 300 µmol/L range and the largest contributions appearing in the upper tail. The smaller male term importance and flatter mid-range shape were consistent with the more heterogeneous male associations seen in the survival analyses.

Proportional hazards diagnostics indicated departures from the proportional hazards assumption in several of the simpler baseline and unlagged time-varying models, particularly in males. By contrast, the lagged and repeated-measure landmark models showed more stable behavior, supporting the interpretation that the more conservative longitudinal specifications provided the most informative estimates.

Exploratory joint longitudinal–survival modeling was undertaken among participants with at least four follow-up SUA measurements (7556 females and 10,832 males). The longitudinal mixed-effects submodels converged, but the current-value and value-plus-slope JMbayes2 joint models did not yield an estimable final survival association parameter. Accordingly, these joint models were considered exploratory and are not interpreted further.

Taken together, the cohort analyses showed the clearest and most consistent positive association in females, whereas the male pattern depended more strongly on how exposure timing and repeated measurement were handled. Analyses that reduced reverse-causation concerns—especially lagged and landmark models—shifted the male signal away from a low-SUA pattern and toward a positive continuous association with higher longitudinal SUA.

## 4. Discussion

In this large EHR-based cohort from Qatar, longitudinal SUA was associated with incident T2DM in a clearly sex-specific manner. The most consistent finding was the female association: higher SUA was associated with higher T2DM hazard in baseline, time-varying, lagged, and landmark analyses, and TWA SUA ranked highly in the interpretable machine learning models. In contrast, the male signal depended more strongly on exposure definition and timing. Simpler unlagged analyses suggested an inverse or low-SUA association, whereas lagged and repeated-measure analyses shifted the male pattern toward a positive association with higher longitudinal SUA.

The female findings are concordant with prospective cohort studies and meta-analyses showing that higher SUA precedes and predicts future T2DM across diverse populations [10,11,12,15]. The magnitude of association in the fully adjusted female baseline model and the similar effect sizes in time-varying and landmark analyses support the interpretation of SUA as a stable risk marker rather than solely a correlate of obesity or concurrent comorbidity.

A key contribution of this study is the explicit use of repeated SUA measurements. Prior longitudinal work suggests that the accumulation and persistence of urate exposure may be particularly relevant to diabetes development [13,16]. In our analysis, repeated-measure and landmark designs improved exposure characterization and reduced the influence of short-term fluctuations. This was especially informative in males, where the apparent excess risk at low SUA attenuated after lagging and with repeated-measure restrictions, while positive continuous associations emerged in the more conservative longitudinal models.

The nonlinear results further refine the clinical interpretation of SUA. In females, both the spline Cox models and the EBM partial-dependence curves suggested that risk begins to increase at comparatively lower SUA levels than in males. This is consistent with prior sex-specific longitudinal evidence suggesting that women may experience dysglycemic risk at lower absolute SUA concentrations, potentially owing to differences in urate handling, renal clearance, and hormonal milieu [14,15,17]. The male J-shaped pattern at baseline also suggests that very low SUA may reflect competing clinical states or reverse-causation pathways that are less apparent once exposure timing is handled more carefully.

Several biological pathways plausibly link elevated SUA to diabetes development. Uric acid has a context-dependent role, functioning as an antioxidant at physiologic levels but potentially acting as a pro-oxidant at higher concentrations, thereby amplifying oxidative stress [5,6,7]. Oxidative stress and inflammatory activation are central mechanisms in insulin resistance and β-cell dysfunction, and SUA-related inflammatory pathways have also been implicated in metabolic syndrome components and related cardiometabolic injury [6,18,19,20].

From a clinical standpoint, SUA is inexpensive and routinely available in primary care. Our findings suggest that longitudinal SUA—particularly when repeatedly elevated—may complement established risk markers such as prediabetes and obesity for diabetes risk stratification. The EBM results further support this interpretation by ranking TWA SUA as one of the more important predictors in the female post-landmark models. However, these observational findings should be interpreted as risk associations rather than evidence that lowering SUA will prevent T2DM. Established prevention strategies, especially lifestyle interventions in high-risk individuals, remain the cornerstone of diabetes prevention [21].

Medication-related findings should be interpreted cautiously. The positive associations involving allopurinol in several models likely reflect confounding by indication, because patients treated with allopurinol may have gout or clinically significant hyperuricemia and may also undergo more intensive surveillance. Similar interpretive challenges have been reported in observational studies of allopurinol exposure and diabetes incidence [22].

Strengths of this study include the large sample size, the high-burden Middle Eastern setting, retention of the full eligible cohort rather than matched subsamples, detailed longitudinal handling of repeated SUA measurements, sex-stratified survival analyses, and triangulation across Cox models, flexible splines, and interpretable machine learning. Limitations include the retrospective observational design, potential residual confounding from lifestyle and socioeconomic factors not captured in the EHR, possible misclassification of comorbidities and medications, and the fact that some simpler models showed departures from proportional hazards. The EBM results are predictive rather than causal, and the exploratory joint models were not estimable.

Overall, higher longitudinal SUA was associated with future T2DM in Qatar, with the clearest positive gradient in females and more exposure-definition-dependent patterns in males. Repeated SUA measurements may therefore help refine sex-specific diabetes risk stratification, while prospective and causal studies remain needed before therapeutic implications can be drawn.

## 5. Conclusions

In conclusion, higher longitudinal SUA was associated with incident T2DM in this Qatar cohort, with consistent positive associations in females and more complex but directionally positive repeated-measure patterns in males. SUA may support enhanced, sex-specific diabetes risk stratification, particularly when characterized using repeated measurements, although causal and therapeutic implications require confirmation in prospective studies.

## Figures and Tables

**Table 1:** Baseline characteristics of the retrospective cohort by sex and sex-specific baseline SUA category (females: low < 149, normal 149–357, and high > 357 µmol/L; males: low < 208, normal 208–428, and high > 428 µmol/L).

Characteristic	Low	Normal	High
Female (*n* = 84,515)			
Participants, *n*	1305	72,071	11,139
Age, years	40.36 (13.05)	43.31 (13.72)	50.89 (15.04)
Baseline SUA, µmol/L	129.72 (19.83)	262.06 (49.39)	409.04 (52.58)
Follow-up, years	3.09 [1.82, 4.73]	3.01 [1.8, 4.69]	2.89 [1.63, 4.67]
Follow-up SUA measurements	1 [1, 2]	1 [1, 2]	2 [1, 3]
Incident T2DM	75 (5.75%)	6200 (8.6%)	1888 (16.95%)
Liver disorder	13 (1%)	423 (0.59%)	101 (0.91%)
Dyslipidemia	312 (23.91%)	20,547 (28.51%)	5182 (46.52%)
Obesity	221 (16.93%)	18,637 (25.86%)	3919 (35.18%)
Chronic kidney disease	10 (0.77%)	1218 (1.69%)	1135 (10.19%)
Prediabetes	98 (7.51%)	11,797 (16.37%)	2627 (23.58%)
Beta-blocker use	57 (4.37%)	4864 (6.75%)	1665 (14.95%)
Statin use	240 (18.39%)	14,319 (19.87%)	4182 (37.54%)
Allopurinol use	5 (0.38%)	621 (0.86%)	1491 (13.39%)
Male (*n* = 85,361)			
Participants, *n*	1639	65,575	18,147
Age, years	52.18 (13.92)	46.67 (13.91)	45.27 (13.15)
Baseline SUA, µmol/L	179.95 (28.31)	339.59 (52.55)	483.25 (51.59)
Follow-up, years	2.86 [1.32, 4.81]	3.13 [1.78, 4.83]	3.14 [1.82, 4.81]
Follow-up SUA measurements	1 [1, 2]	1 [1, 2]	2 [1, 3]
Incident T2DM	364 (22.21%)	7939 (12.11%)	2248 (12.39%)
Liver disorder	28 (1.71%)	818 (1.25%)	180 (0.99%)
Dyslipidemia	887 (54.12%)	25,822 (39.38%)	7248 (39.94%)
Obesity	151 (9.21%)	9117 (13.9%)	4036 (22.24%)
Chronic kidney disease	64 (3.9%)	2389 (3.64%)	1469 (8.1%)
Prediabetes	110 (6.71%)	8498 (12.96%)	3116 (17.17%)
Beta-blocker use	193 (11.78%)	5915 (9.02%)	1821 (10.03%)
Statin use	896 (54.67%)	21,281 (32.45%)	5414 (29.83%)
Allopurinol use	28 (1.71%)	2357 (3.59%)	4995 (27.53%)

**Table 2:** Fully adjusted sex-stratified baseline Cox regression results for incident T2DM.

Sex/Variable	Estimate	Std. Error	Hazard Ratio (95% CI)	*p*-Value
Female (normal SUA = reference)				
High vs normal SUA	0.3666	0.0283	1.44 (1.36–1.53)	<0.001
Low vs normal SUA	−0.1730	0.1163	0.84 (0.67–1.06)	0.137
Per 1 mg/dL increase	0.1492	0.0090	1.16 (1.14–1.18)	<0.001
Age (per year)	0.0202	0.0010	1.02 (1.02–1.02)	<0.001
Obesity (yes)	0.3994	0.0229	1.49 (1.43–1.56)	<0.001
Prediabetes (yes)	0.9348	0.0233	2.55 (2.43–2.67)	<0.001
Male (normal SUA = reference)				
High vs. normal SUA	0.0037	0.0260	1.00 (0.95–1.06)	0.887
Low vs. normal SUA	0.4722	0.0539	1.60 (1.44–1.78)	<0.001
Per 1 mg/dL increase	−0.0425	0.0077	0.96 (0.94–0.97)	<0.001
Age (per year)	0.0165	0.0008	1.02 (1.01–1.02)	<0.001
Obesity (yes)	0.2883	0.0242	1.33 (1.27–1.40)	<0.001
Prediabetes (yes)	0.5173	0.0237	1.68 (1.60–1.76)	<0.001

Adjusted for age, baseline year, liver disorder, dyslipidemia, obesity, chronic kidney disease, prediabetes, beta-blockers, statins, and allopurinol; global PH test *p* < 0.001 in both sexes.

**Table 3:** Comparative sex-specific associations between SUA and incident T2DM across key cohort analyses.

Analysis	Female HR (95% CI)	Female *p*-Value	Male HR (95% CI)	Male *p*-Value
Baseline SUA: high vs normal	1.44 (1.36–1.53)	<0.001	1.00 (0.95–1.06)	0.887
Time-varying current SUA: high vs normal	1.50 (1.41–1.58)	<0.001	0.99 (0.94–1.04)	0.616
365-day lag baseline SUA: high vs normal	1.40 (1.30–1.51)	<0.001	1.19 (1.11–1.28)	<0.001
Landmark TWA SUA (≥2 follow-up measurements): per 1 mg/dL	1.17 (1.13–1.20)	<0.001	1.06 (1.03–1.09)	<0.001

## Data Availability

The data presented in this study are not publicly available because they are subject to institutional privacy and ethical restrictions at Primary Health Care Corporation.

## Supplementary Materials

The following supporting information can be downloaded at: https://www.mdpi.com/article/10.3390/metabo16040251/s1; Figure S1. SHAP decision plot for the female prediction model (n = 300).
