# Supplementary material for: Serum Uric Acid as a Biomarker for Incident Type 2 Diabetes Mellitus: A 6-Year Cohort Study in Qatar
Source: Metabolites. 2026 Apr 8;16(4):251. doi: 10.3390/metabo16040251 (PMC13117883; doi:10.3390/metabo16040251)
Supplement: Supplementary file 1 [file metabolites-16-00251-s001.zip › final_clean_Supplementary_Figure_S1.pdf]

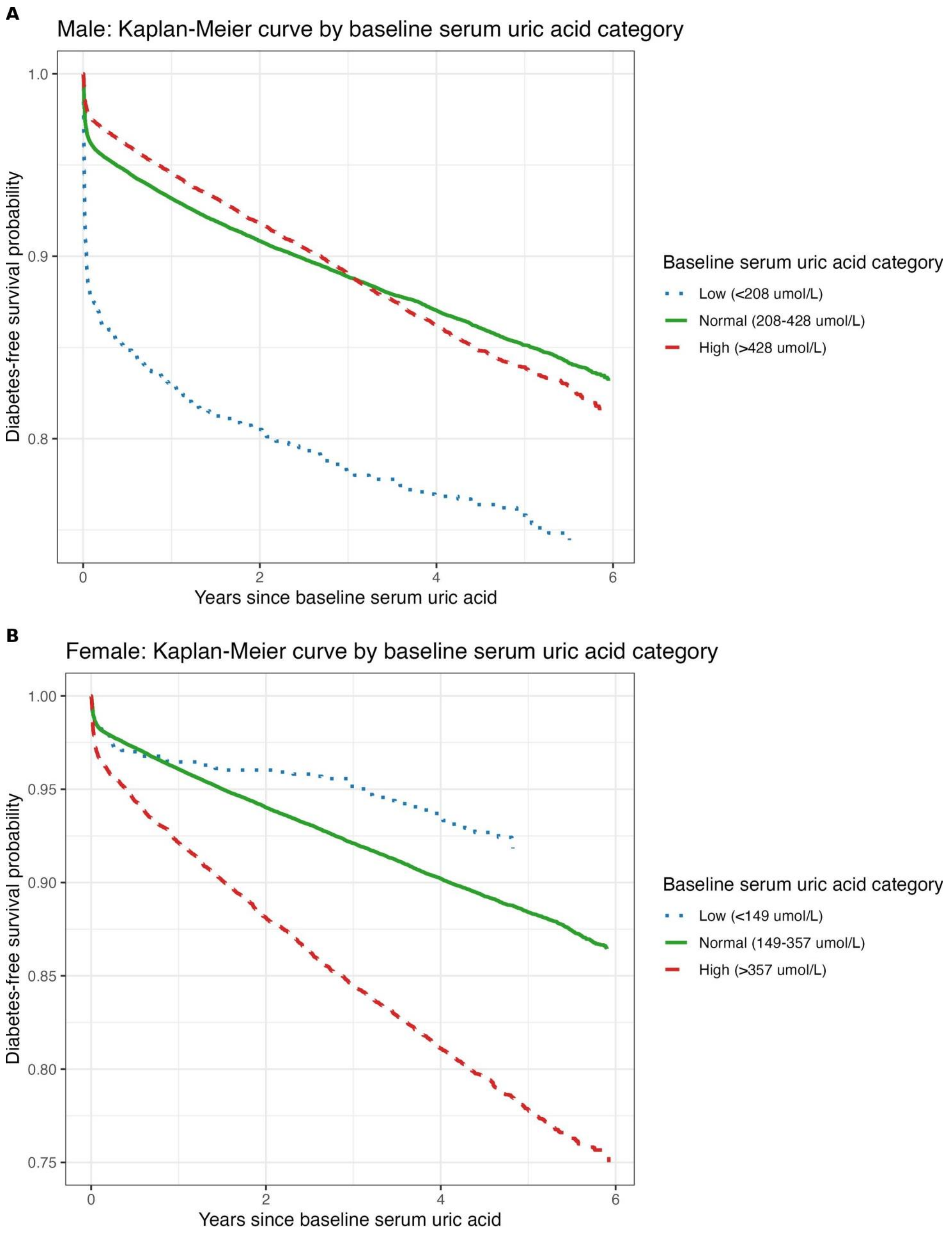
A
Male: Kaplan-Meier curve by baseline serum uric acid category
Diabetes-free survival probability
1.0
0.9
0.8
0
2
4
6
Years since baseline serum uric acid
Baseline serum uric acid category
Low (<208 umol/L)
Normal (208-428 umol/L)
High (>428 umol/L)
B
Female: Kaplan-Meier curve by baseline serum uric acid category
Diabetes-free survival probability
1.00
0.95
0.90
0.85
0.80
0.75
0
2
4
6
Years since baseline serum uric acid
Baseline serum uric acid category
Low (<149 umol/L)
Normal (149-357 umol/L)
High (>357 umol/L)

Supplementary Figure S1. Sex-stratified Kaplan–Meier curves for diabetes-free survival according to baseline serum uric acid (SUA) category. (A) Male participants: low <208 μmol/L, normal 208–428 μmol/L, and high >428 μmol/L. (B) Female participants: low <149 μmol/L, normal 149–357 μmol/L, and high >357 μmol/L. Dotted, solid, and dashed lines denote the low, normal, and high SUA categories, respectively.
